# Detection of Single-Nucleotide and Copy Number Defects Underlying Hyperphenylalaninemia by Next-Generation Sequencing

**DOI:** 10.3390/biomedicines11071899

**Published:** 2023-07-04

**Authors:** Elisabetta Anna Tendi, Giovanna Morello, Maria Guarnaccia, Valentina La Cognata, Salvatore Petralia, Maria Anna Messina, Concetta Meli, Agata Fiumara, Martino Ruggieri, Sebastiano Cavallaro

**Affiliations:** 1 Biomedical Sciences Department, Institute for Biomedical Research and Innovation, National Research Council, Via Paolo Gaifami 18, 95026 Catania, Italy; elisabetta.tendi@irib.cnr.it (E.A.T.); giovanna.morello@irib.cnr.it (G.M.); maria.guarnaccia@cnr.it (M.G.); valentina.lacognata@irib.cnr.it (V.L.C.); 2 Department of Drug and Health Sciences, University of Catania, 95125 Catania, Italy; salvatore.petralia@unict.it; 3 Regional Reference Center for the Treatment and Control of Congenital Metabolic Diseases of Childhood, Department of Clinical and Experimental Medicine, University Hospital Policlinico “Rodolico-San Marco”, 95123 Catania, Italy; messina.marianna@gmail.com (M.A.M.); cmeli@policlinico.unict.it (C.M.); agafiu@virgilio.it (A.F.); 4 Unit of Rare Diseases of the Nervous System in Childhood, Section of Pediatrics and Child Neuropsychiatry, Department of Clinical and Experimental Medicine, University Hospital Policlinico “Rodolico-San Marco”, 95123 Catania, Italy; mruggie@unict.it

**Keywords:** hyperphenylalaninemia (HPA), newborn screening (NBS), diagnosis, targeted next-generation sequencing (tNGS)

## Abstract

Hyperphenylalaninemia (HPA) is the most common inherited amino acid metabolism disorder characterized by serious clinical manifestations, including irreversible brain damage, intellectual deficiency and epilepsy. Due to its extensive genic and allelic heterogeneity, next-generation sequencing (NGS) technology may help to identify the molecular basis of this genetic disease. Herein, we describe the development and validation of a targeted NGS (tNGS) approach for the simultaneous detection of single-nucleotide changes and copy number variations (CNVs) in genes associated with HPA (*PAH*, *GCH1*, *PTS*, *QDPR*, *PCBD1*, *DNAJC12*) or useful for its differential diagnosis (*SPR*). Our tNGS approach offers the possibility to detail, with a high accuracy and in a single workflow, the combined effect of a broader spectrum of genomic variants in a comprehensive view, providing a significant step forward in the development of optimized patient care and management.

## 1. Introduction

Hyperphenylalaninemia (HPA) is the most common autosomal recessive disorder of amino acid metabolism, characterized by defects in the enzymes involved in phenylalanine (Phe) metabolism and resulting in the accumulation of Phe in the blood and other tissues [1]. HPA may result from a deficiency of phenylalanine hydroxylase (PAH), the enzyme catalyzing the conversion of L-Phe to L-tyrosine (Tyr), or from a deficiency of the PAH cofactor, tetrahydrobiopterin (BH4) [2]. The early diagnosis of HPA is essential for the prompt initiation of disease-specific treatment strategies that aim at lowering the blood Phe concentration and include a Phe-restricted diet and/or pharmacologic therapy. In fact, a late diagnosis and untreated HPA usually develop into severe neurological outcomes and a progressive delay in psychomotor development associated with skin rashes, autism, seizures, motor defects, behavioral and psychiatric disorders as well as microcephaly and impaired growth [3,4].

At present, HPA newborn screening (NBS) is performed by measuring both Phe and Tyr and their ratio (Phe/Tyr) level by mass spectrometry (MS/MS) from blood spots. Positive results require confirmation by plasma amino acid analysis and trigger further testing to identify defects in BH4 synthesis and regeneration (pterin levels in the urine and blood, erythrocyte dihydropterin reductase activity) [5]. Although biochemical enzymatic assays are the gold standard for the diagnosis of HPA, genotyping can be recommended for patients and families affected by HPA and may support its management by predicting disease severity, the extent of dietary Phe restriction and response to cofactor (BH4; sapropterin) supplementation [6]. Even though HPA is often caused by genetic defects in the *PAH* gene, a small percentage of cases originate from defects in genes involved in the proper folding of PAH (such as DnaJ heat shock protein family (Hsp40) member C12, *DNAJC12*) or in the biosynthesis and regeneration of BH4 (such as GTP cyclohydrolase I, *GCH1*; 6-pyruvoyl-tetrahydropterin synthase, *PTS*; pterin-4 alpha-carbinolamine dehydratase 1, *PCBD1*; quinoid dihydropteridine reductase, *QDPR*) [3,7]. Mutations in the coding sequence of these genes consist of deletions, duplications, insertions and single-nucleotide variants, which are responsible for various degrees of severity of HPA and severe neurological manifestations such as dystonia, psychomotor retardation and developmental delay [8,9].

The differential diagnosis of HPA should also take into account the genetic investigation of *SPR* (chromosome 2p13.2, three exons) encoding for sepiapterin reductase, an enzyme catalyzing the final step of BH4 biosynthesis, whose defects are responsible for BH4 and neurotransmitter deficiencies without HPA that are difficult to diagnose through traditional NBS [7,10]. In addition, although the lack of SPR has not been associated with HPA in humans, its deficiency in rodents leads to HPA, suggesting that the functional and clinical implications of *SPR* variants should be further investigated for their pleiotropic effects in different species and for their cumulative effects when coupled with other HPA gene variants [11].

In the context of disorders with genic and allelic heterogeneity like HPA, high-throughput technologies such as next-generation sequencing (NGS) are rapidly replacing classic biochemical techniques, allowing the simultaneous detection of several types of genetic alterations (i.e., SNVs and unbalanced structural variants) in multiple genes, helping clinicians to promptly identify the patients’ exact genotype and to optimize patient care and management [12,13,14]. In particular, the implementation of targeted NGS (tNGS) gene panels [15] may be particularly useful for the simultaneous sequencing of multiple genes, offering greater coverage of selected regions of interest, faster turnaround time, manageable interpretation and more clinically relevant data compared to broader genomic profiling, such as whole-exome approaches [16].

In the present study, we describe the development and validation of a tNGS approach for the simultaneous detection of SNVs and copy number variations (CNVs) in genes associated with HPA (*PAH*, *GCH1*, *PTS*, *QDPR*, *PCBD1* and *DNAJC12*) or useful for its differential diagnosis (*SPR*). The integrative approach of multiple genomic variants provides a remarkable opportunity to elucidate the complexity and heterogeneity underlying HPA, expanding the spectrum of disease-relevant variants simultaneously screened in affected individuals and assessing the combinatory effects of such variants across multiple genes.

## 2. Materials and Methods

### 2.1. Sample Collection and DNA Extraction

A total of 38 DNA samples consisting of controls, probands and their parents were recruited by the University Hospital “G. Rodolico—San Marco” in Catania, Italy. The test group consisted of 18 patients (10 females and 8 males), with suspected HPA based on abnormal blood levels of Phe, and 20 controls (18 were parents). The biochemical and clinical parameters of the patients are reported in the Supplementary Materials (S1). This study’s protocol (1327/2020) was approved by the Institutional Review Board, and written informed consent was obtained from all participants or their legal tutors. All methods were performed in accordance with ethical regulations.

Genomic DNA was isolated from dried blood (Guthrie) spots using a QIAamp UCP DNA micro-Kit on an automatic Qiacube extractor (Qiagen, Germantown, MD, USA). The quantification of DNA was assessed by measuring the genomic copies of the human RNase P gene using the TaqMan^®^ RNase P Detection Reagents Kit (ThermoFisher Scientific, Waltham, MA, USA) on the Aria Dx Real-Time PCR system (Agilent Technologies, Santa Clara, CA, USA).

### 2.2. Design of a tNGS Panel

A tNGS panel was designed using the Ion AmpliSeq Designer software (https://ampliseq.com, accessed on 25 May 2020, Pipeline Version 7.43, ThermoFisher Scientific, Waltham, MA, USA) to target 7 genes (*PAH*, *GCH1*, *DNAJC12*, *PTS*, *PCBD1*, *QDPR*, *SPR*) (Table 1) involved in Phe metabolism, as well as the biosynthesis or regeneration of BH4. The panel consists of 56 amplicons (length of 125–275 bp) distributed between two primer pools (28 + 28 primer pairs) and covering a size of 11.38 kb (the complete design of the panel is available in Supplementary Materials S2—Table S1). The in silico coverage was 100%.

### 2.3. Library Preparation and Sequencing

Targeted NGS was performed using the Ion Torrent S5 Platform (ThermoFisher Scientific) according to the manufacturer’s instructions. Genomic DNA libraries were prepared using the Ion AmpliSeq Kit for Chef DL8 (DNA to Library, 8 samples/run) on the Ion Chef System starting from 0.67 ng of input DNA as previously described [17,18]. The standard workflow for library preparation in Ion Torrent sequencing includes fragmentation of the genomic DNA, end-repair of the 3′ and 5′ ends, attachment of specific adapter sequences, size selection of adaptor-ligated molecules and PCR amplification. Specifically, two different linear adapters (A and P1) are ligated to the DNA fragments to incorporate the functionalities required for sequencing. The forward primer was linked with the Ion adapter “A” sequence (5′-CCATCTCATCCCTGCGTGTCTCCGACTCAG-3′) whereas the reverse primer was linked to the Ion adapter “P1” sequence (5′-CCTCTCTATGGGCAGTCGGTGAT-3′). The resulting libraries are ready for the next step in the Ion Torrent sequencing workflow. Samples were distinguished by unique barcodes (Ion Xpress™ Barcode Adapters Kit, ThermoFisher Scientific) and then amplified according to the recommended number of amplification cycles (23 cycles) and annealing/extension time (4 min). Library quality and molarity were assessed using the Ion Library TaqMan Quantitation Kit (ThermoFisher Scientific) on the Aria Dx Real Time PCR System (Agilent Technologies). Serial dilutions of the *E. coli* DH10B Control Library were prepared and run in triplicate to generate a standard curve. The molar concentration of the pooled libraries was determined using the Delta R baseline-corrected raw fluorescence calculated with Aria DX Real-Time PCR Software v 2.0 (Agilent Technologies). The barcoded libraries were super-pooled in a final equimolar concentration of 60 pM each and then loaded on the 510 chip using the Ion 510 Kit on the Ion Chef System. Planned runs in the IonS5 Torrent Suite (v. 5.12.3, ThermoFisher Scientific) had the following parameters: analysis parameters, default; reference library, hg19; target regions, panel; read length, 30×; flows, 550; base calibration mode, default. The plugins used were Coverage Analysis, Ion Reporter Uploader and Variant Caller (default settings).

### 2.4. Computational Analysis of NGS Data

Raw signal data were analyzed using the IonTorrent Suite v.5.12.3 workflow. The pipeline included the quality control of the sequencing run performance assessing Key signal, Test fragment, Read Length, ISP Density and other features related to sequencing.

The sequence data were processed using the Coverage analysis and VariantCaller plugins of the Ion Reporter software version 5.18 (Thermo Fisher Scientific, Waltham, MA, USA). The sequence reads were aligned to the hg19 reference genome, and the variants were prioritized and filtered using the following filter chain on Ion Reporter Software: (a) Filtered Coverage ≥ 100; (b) 50 ≤ Allele Read-Count ≤ 100,000; (c) Location in exonic, intronic, upstream, splicesite_5, splicesite_3; (d) Variant Effect in refAllele, unknown, missense, nonframeshiftInsertion, nonframeshiftDeletion, nonframeshiftBlockSubstitution, nonsense, stoploss, frameshiftInsertion, frameshiftDeletion, frameshiftBlockSubstitution; (e) 0.0 ≤ Minor Allele Frequency ≤ 0.5; (f) a SIFT score less than 0.05 or a PolyPhen-2 score greater than 0.85 were used as reference scores to estimate the evolutionary conservation and the effects of deleterious variants. After the primary data analysis, the filtered variants were manually reviewed in ClinVar, dbSNP build 137, PAH mutation database (PAHvdb, www.biopku.org/home/pah.asp, accessed on 3 March 2023) as well as in the scientific literature. The workflow of the data analysis for NGS is represented in Supplementary Materials S2—Figure S1. SNPs covered by fewer than 250 reads were excluded. The allelic frequencies (AFs) for each SNP were annotated, and each SNP was defined as homozygous when the AF was approximately 100% or 0% and as heterozygous with an AF of approximately 50%.

The Ion Reporter Software was also used to detect CNVs. The CNV detection algorithm was based on a hidden Markov model and used normalized read coverage across amplicons covering the gene to predict copy number or ploidy states. The sample read coverage was compared to a baseline coverage constructed from 10 male control diploid DNA samples. The call of a CNV was made for samples showing a median absolute pairwise difference (MAPD) value <0.5, a metric that measures the read coverage noise detected across all amplicons in a panel to evaluate whether the data can be used for CNV analysis. In addition, the CNV data were filtered to exclude regions with a *p*-value > 10^−5^ and confidence and precision scores lower than 30, as recommended by the manufacturer. Chromosomal gains were associated with a copy number >3 and losses with a copy number <1.

### 2.5. Validation of Variants Identified by NGS

Sanger sequencing was used to validate the variants identified by NGS. Primer pairs were designed using the PrimerBlast tool [19]. The genomic DNA was amplified using specific primers covering the regions of interest. The PCR reactions were performed using the AriaDx Real-Time PCR System (Agilent Technologies), with the following conditions: one cycle at 95 °C for 3 min, 40 cycles of denaturing at 95 °C for 5 s and combined annealing and extension at 60 °C for 10 s. The primer sequences, annealing temperatures and PCR product sizes are listed in Supplementary Materials S3. The PCR amplicons were sequenced by both ends using the Big Dye Terminator v3.1 cycle sequencing kit; the fragments were resolved on a SeqStudio Analyzer, and the results were visualized with Sequencing Analysis Software 7 (ThermoFisher Scientific).

The results obtained through Sanger sequencing were used to evaluate the performance of the NGS workflow and to define the accuracy, sensitivity and specificity. True-positive (TP), true-negative (TN), false-positive (FP) and false-negative (FN) variant calls were defined by considering the results from the Sanger sequences analysis obtained from the probands. Specifically, TP variants were detected by both the NGS filtering pipeline and Sanger sequencing. TN variants (reference nucleotides) were called neither by NGS nor by Sanger sequencing. The FPs were variants detected by NGS but not by Sanger sequencing. The FNs were variants detected by Sanger sequencing but missed by NGS. The accuracy was calculated as follows: (TP + TN)/(TP + FP + TN + FN); the sensitivity was calculated as follows: TP/(TP + FN); and the specificity was calculated as follows: TN/(TN + FP).

### 2.6. CNV Validation

CNVs detected by the tNGS analysis were confirmed by quantitative PCR. Primers for CNV validation were designed using the Primer-BLAST software tool [19]. Briefly, *RPPH* was used as an endogenous control gene for normalization. Reactions were performed using the Brilliant III Ultra-Fast SYBR^®^ Green qPCR Master Mix (Agilent Technologies), which included an internal reference (ROX). Each qPCR reaction comprised 2 ng DNA, 10 μL Brilliant SYBR Green QPCR Master Mix and forward and reverse primers at the final concentration of 10 μM. The qPCR reactions were performed using the AriaDx Real-Time PCR System (Agilent Technologies). The reaction profile was one cycle at 95 °C for 3 min, 40 cycles of denaturing at 95 °C for 5 s and combined annealing and extension at 60 °C for 20 s, and one cycle at 95° for 1 min, 60 °C for 30 s and 95 °C for 30 s. Each qPCR experiment was run in triplicate. The DNA samples were analyzed with the test and reference primers on the same reaction plate. The relative quantification was measured using the ∆∆Ct method, which requires a healthy control sample (diploid) as a calibrator in all amplifications. Moreover, 2-ΔΔCt ≥ 1.4 or ≤0.6 was defined as copy number gain or loss, respectively, whereas 2-ΔΔCt values from 0.8 to 1.2 were considered normal diploid. The PCR products were visualized using agarose gel electrophoresis. The primer sequences, annealing temperatures and PCR product sizes are shown in Supplementary Materials S3.

## 3. Results

### 3.1. Performance and Coverage Analysis

In this work, we developed and validated a tNGS approach for the analysis of SNPs, insertions/deletions or chromosomal aberrations in seven genes (*PAH*, *DNAJC12*, *GCH1*, *PTS*, *SPR*, *PCBD1*, *QDPR*) implicated in Phe metabolism (the complete design of the NGS panel is available in Supplementary Materials S2, Table S1). The efficiency and validity of the NGS panel were evaluated by testing 38 DNA samples, including controls, probands and parents. The samples were processed by NGS with the minimum sequencing depth set to 100×. In this configuration, the median coverage was 1000×, with 100% of amplicons with >250× coverage. All variants met the phred-scaled quality score, Q ≥ 30.

**Figure 1:**
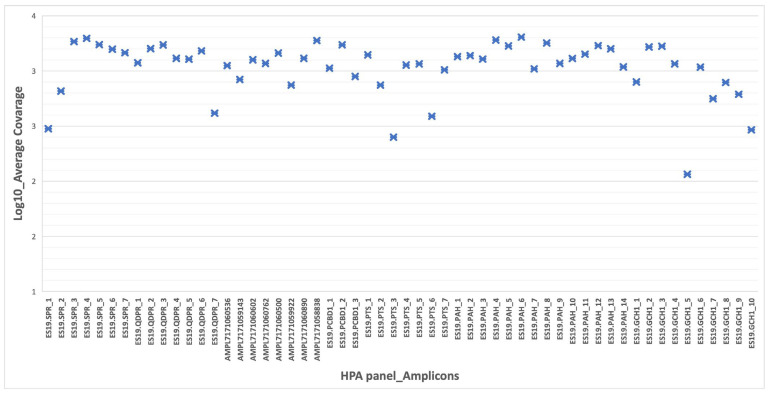
CNVs identified by tNGS in HPA patients.

In the run metrics’ results, all samples were uniformly covered at depths that exceeded the minimum coverage required (30×) for the accurate calling of variants. Adequate amplification efficiency was observed for all amplicons (100%) (mean assigned read per amplicon Log10 ranging from 2 to 3.5) (Figure 1). The overall accuracy, specificity and sensitivity of the NGS panel was 100%.

### 3.2. Identified Pathogenic Variants

The identification of genomic variation is a crucial step for unraveling the relationship between genotype and phenotype and obtaining important insights in routine research and clinical settings. After the assessment of quality metrics, the variants filtered according to a stepwise strategy were manually curated and reviewed in the ClinVar database, the dbSNP build 137, the PAH mutation database and the PAHvdb database as well as in the scientific literature to exclude nonpathogenic variants. The analytic pipeline included the assessment of point mutations and copy number alterations. Starting with 227 variants listed in Variant Caller (IonTorrent Suite v.5.12.3) related to 18 probands, our filtering pipeline identified 17 different PAH pathogenic variants, combined in different genotypes (Table 2).

The analyzed patients were mainly compound heterozygous (17/18), and one was homozygous (c.782G>A; c.782G>A). The mutation types were as follows: mostly missense (12/17), splice-site (1/17), frameshift deletions (1/17), nonsense mutations (2/17) and unknown (1/17). The most frequent mutation was c.1066-11G > A, followed by c.898G>T, c.1208C>T and c.782G>A. No mutations were found in the other genes present in the NGS panel. The effect of variants on the PAH enzyme activity depends on the position and nature of the variant, which determines the phenotype of the patient. Table 3 shows the correlation, in our cohort, between PAH variants and blood Phe levels ranging from 115 to 1020 μM.

### 3.3. CNVs in Targeted Genes

**Figure 2:**
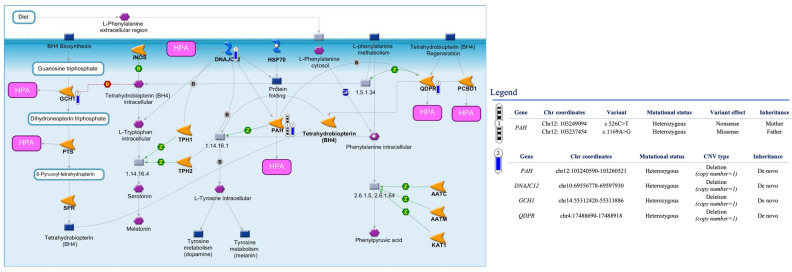
Integrative analysis of multiple HPA-related genomic alterations identified in proband #8. The interaction pathway map represents the functional correlation between multiple genomic data types (CNVs, SNVs) affecting multiple HPA-related genes in proband #8 of our patient cohort. The map was created using the MetaCore Pathway Map Creator tool (GeneGo). The experimental data are shown on the maps as “thermometer-like” figures. In particular, the thermometers with black/white stripes and #1 represent the PAH genomic variants, while the CNV deleted regions are presented on the map as “thermometer” #2 and labeled with downward thermometers (blue). Further explanations are provided at https://portal.genego.com/help/MC_legend.pdf (accessed on 3 March 2023).

**Figure 3:**
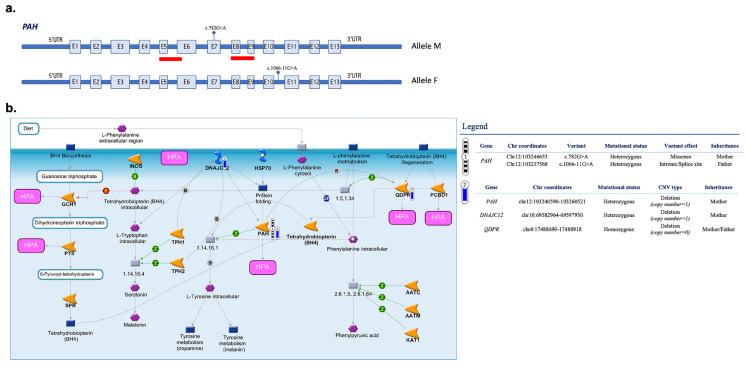
Integrative analysis of multiple HPA-related genomic alterations identified in proband #16. (**a**) Schematic representation of the *PAH* gene and the pathogenic variants that we found in proband #16, including the pathogenic deletion encompassing exon 5 and exons 8–9 transmitted by maternal inheritance (Allele M) and occurring in *cis* with the c.782G>A and in *trans* with 1066-11G>A (Allele F, paternal inheritance). (**b**) The interaction pathway map represents the functional correlation between multiple genomic data types (CNVs, SNVs) affecting multiple HPA-related genes in proband #16 of our patient cohort. The map was created using the MetaCore Pathway Map Creator tool (GeneGo). The experimental data are shown on the maps as “thermometer-like” figures. In particular, the thermometers with black/white stripes and #1 represent the PAH genomic variants, while the CNV deleted regions are presented on the map as “thermometer” #2 and labeled with downward thermometers (blue). Further explanations are provided at https://portal.genego.com/help/MC_legend.pdf (accessed on 3 March 2023).

Six deletions showing an overlap with genomic alterations previously classified as pathogenic in ClinVar for HPA/PKU were detected by tNGS in five HPA-related genes (Table 4). Among these variations, we found a hemizygous deletion encompassing exon 5 and exons 8–9 of *PAH* in two unrelated probands (Table 4) [20]. This deletion was detected as a de novo variant in proband #8, who also carries two bi-allelic pathogenic variants in other *PAH* exons (Figure 2, Table 3 and Table 4). Proband #16 showed the same pathogenic deletion transmitted by maternal inheritance and occurring in *cis* with the c.782G>A (p.R261P; pathogenic in ClinVar) and in *trans* with the pathogenic intronic variant 1066-11G>A previously associated with a classical HPA-deficiency phenotype (Figure 3a, Table 3 and Table 4). Of note, both these probands showed additional pathogenic deletions in other HPA-related genes, including the full or partial deletion of *DNAJC12* [21] and the deletion of exon 7 in *QDPR*, which was observed in the heterozygous or homozygous state (Figure 2 and Figure 3, Table 4). In addition, a gross deletion of the genomic region encompassing exons 4–5 of *GCH1* [22] was observed in proband #8 (Figure 2, Table 4). The deletion of *SPR* exon 1, which includes the initiator codon and is expected to result in an absent or disrupted protein product [23], was identified in proband #9 (Table 4).

## 4. Discussion

The early diagnosis and management of genetic conditions in newborns to improve patient outcomes is one of the main goals of the healthcare system as stated by the American College of Medical Genetics (ACMG) [24]. Among the genetic disorders affecting millions of children worldwide, altered Phe metabolism has been included in NBS programs. The detection of Phe levels using biochemical NBS tests and tandem mass spectrometry on a blood spot is considered the gold standard for the diagnosis of symptomatic HPA patients. However, these methods do not discriminate between the diverse phenotypes, thus, not allowing appropriate treatment in a timely manner [3]. Sequencing technology advances and, in particular, custom-designed tNGS panels are poised to improve diagnostics, representing a better choice for the simultaneous screening of multiple HPA-related genes and different types of genetic variants (including SNVs and CNVs) with a high sequencing capacity and broad coverage. Previous studies investigated the clinical utility of NGS to diagnose PKU and other forms of HPA, but they failed to evaluate the contribution of CNVs [25].

The aim of this study was to develop and validate a tNGS approach to genotype patients with HPA through the simultaneous detection of sequence alterations and CNVs in seven genes implicated in Phe metabolism (*PAH*, *GCH1*, *PTS*, *QDPR*, *PCBD1*, *DNAJC12*, *SPR*). To evaluate its clinical utility, we performed NGS studies using DNA from blood collected from a Guthrie card, which can therefore be used for both biochemical and genomic investigations. It is well known that gene variants can differently affect the enzymatic activity of PAH, resulting in different metabolic phenotypes [26]. Over 1500 *PAH* variants are currently known (PAHvdb: Phenylalanine Hydroxylase Gene Locus-Specific Database, http://www.biopku.org/home/pah.asp; accessed on 3 March 2023), and depending on the inherited alleles, affected individuals may have a very mild to a pronounced increase in Phe [27]. Concerning the type of pathogenic variants found in our cohort, our tNGS approach was able to identify 17 different *PAH* SNPs, including unknown, nonsense, splice, frameshift deletion and, mostly, missense, with the latter causing protein misfolding and/or impairing catalytic function [28]. Below is a detailed description of these 17 variants and their effect on the PAH enzymatic activity according to the public databases and the scientific literature data.

In our cohort, the most prevalent *PAH* variant was the intronic c.1066-11G>A (p.Gln355_Tyr356insGlyLeuGln, IVS10-11G>A), which is predicted to affect normal splicing and to lead to 0% enzyme activity (http://www.biopku.org/centralStore/biopku/PAH%20activity.pdf; accessed on 3 March 2023). The amount of PAH protein in the liver of homozygous patients is normal regardless of the absence of catalytic activity, with the latter probably caused by the insertion of three additional amino acids (Gly-Leu-Gln) between the normal sequences encoded by exon 10 and exon 11, leading to conformational changes [29] This is one of the most common pathogenic variants causing PKU associated with both a classical and a moderate PKU phenotype in patients carrying a second variant in *PAH* [30]. Since this variant has a high frequency in the Mediterranean area, it was renamed the “Mediterranean variant” [31]. The second most common variant found in this study was c.898G>T (p.Ala300Ser), which represents the most common pathogenic variant found in patients with PKU [32,33], being mostly associated with a milder phenotype and BH4 responsiveness [34]. The milder phenotype is correlated to the 31% enzyme activity retained by the p.Ala300Ser variant [35]. The c.121C>T (p.Leu41Phe) variant has been associated with 10% total enzyme activity [36]. The c.143T>C (p.Leu48Ser) variant has been reported in multiple individuals with mild and classic PKU (BH4 deficiency excluded). Experimental studies have shown that this missense change causes low PAH enzymatic activity [33,37]. The c.165delT (p.Phe55fs) frameshift variant is a single-base deletion in *PAH* exon 2. This mutation causes a frameshift generating a stop signal (TAA), which results in an absent or disrupted protein product with a residual activity of 0% and increased Phe plasma levels. This premature translational stop signal has been reported in the compound heterozygote state in association with HPA and/or the classic PKU phenotype [37,38]. The c.442-5C>G mutation occurs in intron 4 of *PAH* and has been reported in combination with another *PAH* variant in an individual with mild PKU [38]. Prediction analysis suggests that this variant may disrupt the consensus splice site. The c.526C>T (p.Arg176Ter) variant creates a premature translational stop signal at codon 176 in *PAH* resulting in an absent or disrupted protein product. This particular variant has been reported in several individuals affected with HPA and, in the homozygous state, is associated with classic PKU [39]. The c.781C>T (p.Arg261Ter) variant transforms Arg261 to a stop codon in exon 7. The c.782G>A (p.Arg261Gln) variant is associated with classic or moderate PKU [32,38], showing 15.5–30% of PAH activity [33,40]. The c.842C>T (p.Pro281Leu) variant is located at an intron/exon boundary and, in vitro, was shown to abolish the function of PAH [26]. The c.848T>A missense variant, located on exon 8, replaces isoleucine with asparagine at codon 283 of *PAH* [33,41]. Multiple affected individuals carry this variant in *trans* with known pathogenic variants [32,42]. The c.1028A>G (p.Tyr343Cys) variant results in a nonconservative amino acid change located in the aromatic amino acid hydroxylase, C-terminal domain of the encoded protein sequence [32]. The c.1045T>C (p.Ser349Pro) variant replaces serine with proline at codon 349 of *PAH* altering the function of its encoded protein. This variant is associated with a severe phenotype and has been reported as not responsive to BH4 therapy [35]. The c.1139C>T (p.Thr380Met) variant is associated with mild HPA and is the most frequent one in Italian patients. Located on exon 11, this variant affects the PAH catalytic domain and reduces its activity (>25%) [43]. c.1169A>G (p.Glu390Gly) is a missense variant classified as BH4 responsive [34,35,44] and causes reduced (42–62%) PAH activity [40,44,45]. The c.1208C>T (p.Ala403Val) missense variant, classified as BH4 responsive, is associated with mild HPA [31] and residual PAH activity (12–32%) [35]. Finally, the c.1241A>G variant, associated with BH4 responsiveness, results in an amino acid change (p.Tyr414Cys) affecting the structure/function of PAH [28,35]. It has been reported in a homozygous or compound heterozygous state with other pathogenic variants [37,46].

In addition to the SNVs, our tNGS panel identified a number of single-exon/multiexon deletions in multiple HPA-related genes, allowing the evaluation of the combined effect of these alterations in a comprehensive and detailed view. We found an intragenic deletion involving exons 5, 8 and 9 of the *PAH* gene in two probands (#8 and #16) of our patient cohort that overlapped with CNVs previously described in HPA patients [25]. These deletions may disrupt the reading frame and alter regions that are critical to PAH function. Together with other pathogenic variants in the same or other HPA-related genes (Figure 2 and Figure 3), these variants may contribute to HPA. An interesting example is represented by proband #16 showing the co-occurrence of *PAH* multi-exon deletion and two *PAH* SNVs (c.782G>A in *cis* and 1066-11G>A in *trans*) together with likely-pathogenic deletions of *DNAJC12* and *QDPR* (Figure 3). Although alterations in *DNAJC12* and *QDPR* have been associated with various symptoms such as hypotonia, dystonia, parkinsonism/hypokinetic rigid syndrome and progressive motor and cognitive developmental disorders, these comorbidity phenomena were not reported in our patient. Similarly, proband #8 had SNVs in *PAH* and chromosomal aberrations in *DNAJC12*, *QDPR* and *GCH1* (Table 3) but showed normal biochemical values for pterins (neopterin and biopterin) and QDPR enzymatic activity without relevant clinical manifestations [47]. In these cases, therefore, the presence of complex genomic variants was not clearly distinguishable at the phenotypic level.

This study is the first to apply an integrated analysis that combines SNPs, CNVs and other types of genomic variants that, alone or in combination, may concur to determine different HPA phenotypes. The results obtained demonstrated that our tNGS approach can identify, with high accuracy and in a single workflow, a broader spectrum of genomic variants whose presence is currently underestimated. When compared to other traditional screening methods, such as Sanger sequencing, our tNGS approach also has advantages in terms of cost and time [3]. The adoption of this approach in gene testing may produce a turning point in the clinical diagnosis of HPA, allowing a more comprehensive understanding of the complex and heterogeneous basis of the disease, optimizing clinical management, reducing the psychological burden and improving the development of early and effective therapeutic interventions.

## Figures and Tables

**Table 1:** NGS targeted custom panel designed with AmpliSeq Designer Tool.

Gene	Cytogenetic Location	Number of Exons	Number of Amplicons	Pathology	Phenotype
					OMIM No.
*PAH*	12q23.2	13	14	Phenylketonuria (homozygous)	261600
				Hyperphenylalaninemia, non-PKU mild (heterozygous)	
*DNAJC12*	10q21.3	6	8	Hyperphenylalaninemia, mild, non-BH4-deficient	617384
*QDPR*	4p15.32	7	7	Hyperphenylalaninemia, BH4-deficient, C	261630
*PTS*	11q23.1	6	7	Hyperphenylalaninemia, BH4-deficient, A	261640
*PCBD1*	10q22.1	6	3	Hyperphenylalaninemia, BH4-deficient, D	264070
*GCH1*	14q22.2	7	10	Dystonia, DOPA-responsive, with or without hyperphenylalaninemia	128230
				Hyperphenylalaninemia, BH4-deficient, B	233910
*SPR*	2p13.2	3	7	Dystonia, dopa-responsive, due to sepiapterin reductase deficiency	612716

**Table 2:** PAH pathogenic variants identified by NGS in 18 patients.

Nucleotide Aberration	Protein Change	Location	Type	dbSNP	Probands Carrying Variant
c.121C>T	L41F	E2	Miss	rs62642928	1
c.143T>C	L48S	E2	Miss	rs5030841	1
c.165delT	F55fs	E2	Del	rs199475566	1
c.442-5C>G	-	I4	Unkn	rs62514909	1
c.526C>T	R176 *	E6	Non	rs199475575	2
c.781C>T	R261 *	E7	Non	rs5030850	1
c.782G>A	R261Q	E7	Miss	rs5030849	4
c.842C>T	P281L	E7	Miss	rs5030851	1
c.848T>A	p.Ile283Asn	E8	Miss	rs62508693	1
c.898G>T	A300S	E8	Miss	rs5030853	5
c.1028A>G	Y343C	E10	Miss	rs62507265	1
c.1045T>C	S349P	E10	Miss	rs62508646	1
c.1066-11G>A	-	I10	Splic	rs5030855	7
c.1139C>T	T380M	E11	Miss	rs62642937	1
c.1169A>G	E390G	E11	Miss	rs5030856	2
c.1208C>T	A403V	E12	Miss	rs5030857	4
c.1241A>G	Y414C	E12	Miss	rs5030860	1

Reference sequence: NM_000277.3; Non: nonsense; Miss: missense; Del: deletion; Splic: splicing; I: intron; E: exon; Unkn: unknown; * Stop codon.

**Table 3:** Correlation between PAH variants and blood Phe levels in 18 patients.

Proband	Sex	Age at On-Set/First Event	Phe Blood Conc. (μM) at Diagnosis	Detected Mutation			Detected Mutation		
				Systematic Name	FI	MI	Systematic Name	FI	MI
#1	M	birth	726	c.782G>A	X		c.782G>A		X
#2	F	birth	678	c.842C>T	n.a.	n.a.	c.1028A>G	n.a.	n.a.
#3	M	birth	119	c.442-5C>G	n.a.	n.a.	c.1208C>T	n.a.	n.a.
#4	F	birth	1117	c.1066-11G>A	n.a.	n.a.	c.165delT	n.a.	n.a.
#5	M	birth	400	c.1066-11G>A	n.a.	n.a.	c.143T>C	n.a.	n.a.
#6	F	birth	154	c.1241A>G		X	c.1139C>T	X	
#7	F	birth	193	c.898G>T	X		c.1066-11G>A		X
#8	F	birth	514	c.526C>T		X	c.1169A>G	X	
#9	M	birth	115	c.1169A>G	n.a.		c.1208C>T		X
#10	M	birth	194	c.898G>T	X		c.1066-11G>A		X
#11	M	birth	363	c.526C>T	n.a.	n.a.	c.781C>T	n.a.	n.a.
#12	F	birth	1930	c.121C>T	n.a.	n.a.	c.782G>A	n.a.	n.a.
#13	F	birth	1271	c.1045T>C		X	c.1066-11G>A	X	
#14	F	birth	173	c.782G>A	X		c.898G>T		X
#15	M	birth	224	c.898G>T		X	c.1066-11G>A	X	
#16	F	birth	701	c.782G>A		X	c.1066-11G>A	X	
#17	M	birth	149	c.898G>T		X	c.1208C>T	X	
#18	F	birth	242	c.848T>A		X	c.1208C>T	n.a.	

FI: father inheritance; MI: mother inheritance; n.a.: not available.

**Table 4:** CNVs identified by tNGS in HPA patients.

Locus	Length	Gene	CNV	Exon(s)	Functional Interpretation (ClinVar)	Proband(s)
chr 2:73114493-73114894	401	*SPR*	Heterozygous	1	Pathogenic (SPR deficiency; Dopa-responsive dystonia)	#9
			Deletion			
chr12:103240590-103260521	19,931	*PAH*	Heterozygous	5, 8–9	Pathogenic	#8, #16
			Deletion		(HPA)	
chr10:69556778-69597930	41,152	*DNAJC12*	Heterozygous	4–5	Pathogenic	#8
			Deletion		(HPA, mild, non-BH4-deficient)	
chr10:69582964-69597930	14,966	*DNAJC12*	Heterozygous	1–2	Pathogenic	#8, #16
			Deletion		(HPA, mild, non-BH4-deficient)	
chr14:55312420-55313886	1466	*GCH1*	Heterozygous	4–5	Pathogenic	#8
			Deletion		(GTP cyclohydrolase I deficiency)	
chr4:17488690-17488918	228	*QDPR*	Heterozygous/Homozygous Deletion	7	Uncertain significance (BH4-deficient HPA)	#8, #16

## Data Availability

Not applicable.

## Supplementary Materials

The following supporting information can be downloaded at https://www.mdpi.com/article/10.3390/biomedicines11071899/s1, Supplementary Materials S1: Clinical characteristics of PAH samples; Supplementary Materials S2: Complete design of the HPA panel; Supplementary Materials S3: Variant validation: primer sequences, annealing temperatures and PCR product sizes.
